# Validation of a Fall Predictive Model for Inpatients in Japanese Long Term Care Hospitals

**DOI:** 10.7150/ijms.106600

**Published:** 2025-06-09

**Authors:** Hitomi Shimada, Risa Hirata, Naoko E. Katsuki, Eiji Nakatani, Kiyoshi Shikino, Maiko Ono, Midori Tokushima, Tomoyo Nishi, Shizuka Yaita, Chihiro Saito, Kaori Amari, Kazuya Kurogi, Yoshimasa Oda, Mariko Yoshimura, Shun Yamashita, Yoshinori Tokushima, Hidetoshi Aihara, Motoshi Fujiwara, Masaki Tago

**Affiliations:** 1 Department of General Medicine, Saga University Hospital, Saga, Japan.; 2 Shimada Hospital of Medical Corporation Chouseikai, Saga, Japan.; 3 Department of Biostatistics and Health Data Science, Graduate School of Medical Science Nagoya City University, Nagoya, Japan.; 4 Department of General Medicine, Chiba University Hospital, Chiba, Japan.; 5 Department of Community-oriented Medical Education, Chiba University Graduate School of Medicine, Chiba, Japan.; 6 Department of General Medicine, Karatsu Municipal Hospital, Saga, Japan.; 7 Shizuoka General Hospital, Shizuoka, Japan.; 8 Clinical Education Center, Saga-Ken Medical Centre Koseikan, Saga, Japan.; 9 Department of General Medicine, National Hospital Organization Ureshino Medical Center, Saga, Japan.; 10 Department of General Medicine, Yuai-Kai Foundation and Oda Hospital, Saga, Japan.; 11 Safety Management Section, Saga University Hospital. Saga, Japan.

**Keywords:** accidental falls, long-term care hospital, Japan, aged, fall risk model, inpatients

## Abstract

***Background:*** The Saga Falls Risk Model 2 (SFRM2) is a simplified fall prediction model that we recently developed. It uses eight items that are easy to assess at the time of admission to an acute care hospital. However, patients in long-term care hospitals have poor activities of daily living and a high risk of falls compared to those in acute care hospitals. Although effective fall predictive models exist for long-term care hospitals, their accuracy remains suboptimal. This study aimed to validate the SFRM2 for predicting falls in long-term care hospital patients.

***Methods:*** This multicenter retrospective observational study was conducted in three long-term care hospitals in Japan from April 2018 to March 2021. All inpatients aged ≥20 years were included. The eight items of the SFRM2 (age, sex, emergency admission, department of admission, hypnotic medication use, history of falls, eating independence, and Bedriddenness rank) and in-hospital falls were collected from medical records. The accuracy of SFRM2 was assessed by calculating the area under the curve (AUC) and shrinkage coefficient, as well as the sensitivity, specificity, positive predictive value, and negative predictive value.

***Results:*** Among the 1182 patients (median age: 86 years, 538 males) included in the analysis, 140 (11.8%) experienced in-hospital falls. The fall incidence rate was 4.4 per 1000 patient-days. SFRM2 exhibited an AUC of 0.889 (95% confidence interval: 0.861-0.916), consistent with the actual incidence of falls, with a shrinkage coefficient of 0.975. The cutoff score for SFRM2 on the Youden index was -2.14, with a sensitivity of 77.9%, specificity of 84.7%, positive predictive value of 40.6%, and negative predictive value of 96.6%.

***Conclusion:*** SFRM2 showed good discriminative ability in external validation at long-term care hospitals. Its applicability in this setting may be advantageous due to the relatively stable condition of older inpatients compared to those in acute care hospitals.

## Background

Recently, fall injuries in aging populations have become a concern in Japan. Fall injuries have been shown to increase with advancing age, with an incidence of 5.5% per year among individuals aged 65-79 years compared to 9.5% among those aged ≥85 years 1. In Japan, falls and fractures resulting from a fall account for 12.0% of new long-term care insurance users annually 2. A recent review on falls by McKercher et al. advocates for comprehensive fall assessment for older adults 3. Developing accurate fall predictive models for older adults is therefore crucial for identifying high-risk individuals in this aging society and implementing preventive interventions, thereby reducing healthcare costs.

The Saga Fall Risk Model 2 (SFRM2) was developed as a fall prediction model for adult inpatients in acute care hospitals by assessing eight parameters: age, sex, emergency admission, department of admission, hypnotic medication use, history of falls, eating independence, and Bedriddenness rank (BR) 4. While other fall predictive models, including the Hendrich II Fall Risk Model (HFRM II) and Morse Fall Scale, have been developed and validated, they are often complex and impractical in busy Japanese hospitals 5-7. According to fall prevention guidelines by Manuel Montero-Odasso et al., the use of fall-risk screening tools is not recommended as they do not reduce falls and waste valuable time 8. However, SFRM2 only utilizes eight parameters and does not require special evaluation skills or testing, making it suitable for admission assessment 4. Our previous studies have validated SFRM2 in acute care and university hospitals, demonstrating high accuracy 9,10. However, a validation study in an acute-care hospital with long-term care beds reported an area under the curve (AUC) of 0.640 for SFRM2 11, suggesting the need for further validation.

In Japan, 77.5% of inpatients in long-term care hospitals require medical care (e.g., management of chronic diseases, frequent sputum suctioning, 24-hour monitoring), and 83.6% require nursing assistance or are bedridden 12. Moreover, the Barthel index of fallen patients is significantly lower than that of non-fallen patients 11, indicating a high risk of falls in patients with poor activities of daily living (ADLs) requiring nursing care. The fall rate of patients in long-term care beds is higher than that in previous studies conducted in acute-care hospitals 13. Existing fall predictive models for overseas hospitals with similar backgrounds to Japanese long-term care hospitals show limited accuracy (AUCs of approximately 0.6) 14,15. Therefore, evaluating the accuracy of SFRM2 in predicting falls among long-term care patients at high risk can aid in reducing fall-related problems. This study aimed to clarify the accuracy of SFRM2 through external validation using data from several long-term care hospitals in Japan.

## Methods

### Study design, setting, and participants

This multicenter retrospective observational study included all inpatients aged ≥20 years from three Japanese long-term care hospitals from April 2018 to March 2021. Background information on the three hospitals is provided in S1. Patients with missing SFRM2 data were excluded.

### Data and definitions

Data were extracted from medical records. The following information was collected: date of admission, age at admission 16,17, sex 16-18, department of admission (internal medicine or others) 19, emergency admission (present or absent), emergency transport (present or absent), referral letter (present or absent), BR and Cognitive function scores (CFS) according to the Ministry of Health, Labour and Welfare (MHLW) of Japan 20, hypnotic medication use at admission (present or absent) 18, history of falls (present or absent) 18,21, operations during hospitalization 22, rehabilitation (present or absent) 19,23, in-hospital falls (present or absent), date of discharge, eating independence (independent, requiring assistance), visual impairment 17, and primary disease during hospitalization.

Regarding the department of admission, none of the patients were admitted to the neurosurgery department. Additionally, based on past medical records, almost all patients were admitted to internal medicine. Thus, patients were classified as being admitted under internal medicine or others in this study. BR and CFS are official ADL indicators used in Japanese medical and nursing care settings, as proposed by the MHLW 24. BR is classified into five major and nine detailed categories, whereas CFS is classified into six major and eight detailed categories. This study evaluated BR in five major categories (normal, J: independence/autonomy, A: house-bound, B: chair-bound, or C: bed-bound) and CFS in six major categories (normal, I, II, III, IV, M). Similar to our previous studies, benzodiazepines and non-benzodiazepines were included among hypnotic medications, except for melatonin receptor agonists and orexin receptor antagonists 4. The length of stay was calculated based on the dates of admission and discharge. Falls were defined as any unexpected fall from any height or position, including falls from stairs, chairs, beds, walking, sitting, or lying down, regardless of injury. Data on the first in-hospital fall was collected from medical records, while the history of falls was collected from incident and accident reports.

### Statistical analysis

The primary outcome of this study was the first in-hospital fall, excluding the occurrence of subsequent falls. Patients who met the inclusion criteria were divided into two groups: a fall group (at least one in-hospital fall) and a non-fall group. Descriptive statistics for survey items were presented as continuous (medians with interquartile range) and categorical variables (absolute numbers with percentages). Comparisons between groups were calculated using the Mann-Whitney U test for continuous variables and the chi-squared test for categorical variables. Multiple testing correction was not considered for exploratory analysis.

The algorithm for the SFRM2, is as follows: SFRM2 = -5.8563 + 0.0096 × (Age) + (Male = 0.5684) + (Emergency admission = 0.4418) + (Admitted department; Neurosurgery = 0.6520) + (Hypnotics; Using = 0.3612, Missing data = 0.2139) + (History of fall = 0.4362) + (Ability of eating; Independent = 0.2352, Missing data = -1.0436) + (BR; J = 1.3758, A = 1.8317, B = 1.9186, C = 1.7205, Not assessable = -0.1505).

Model accuracy was determined by calculating the AUC, 95% confidence interval (CI), and shrinkage coefficient for the scores of each inpatient. Three cutoff points were utilized: the minimum score with 90% sensitivity, the optimal score based on the Youden index, and the maximum score with 90% specificity. Multivariable logistic regression analysis was conducted using forced entry of all eight SFRM2 parameters to predict the first in-hospital fall. All statistical analyses were performed using SPSS Statistics version 27 (IBM), and statistical significance was set at p <0.05.

### Sample size

The required sample size of 250 cases was based on the AUC of SFRM2 in the previous study 4,11, assuming an effect size of 0.20 (predicted AUC: 0.70, null hypothesis AUC: 0.50), an estimated fall rate of 7.1%, an alpha error of 0.05, and a beta error of 0.20.

## Results

### Participant backgrounds and incidence of fall events

**Figure 1:**
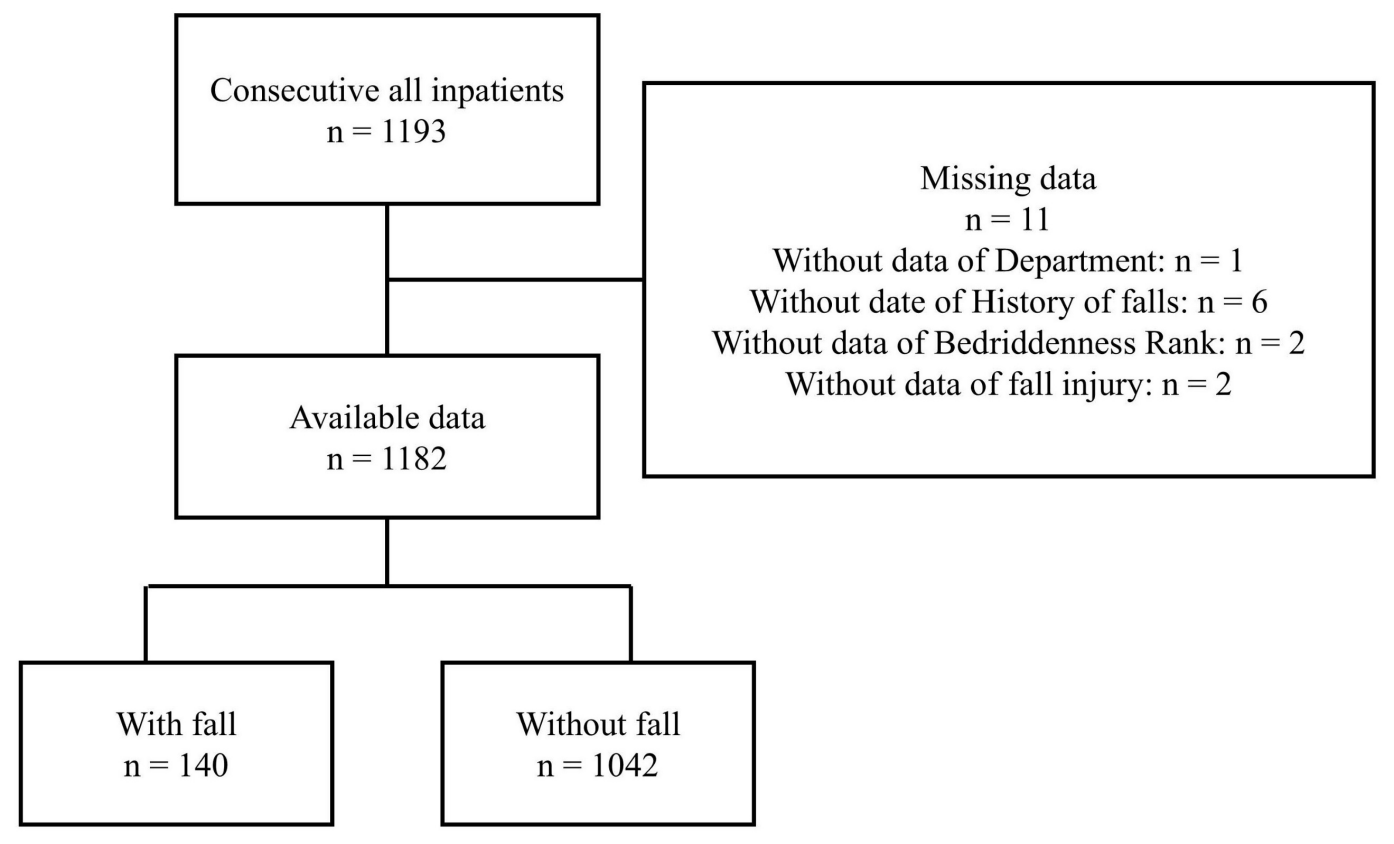
Data flow diagram. A total of 1182 participants were included, and 140 falls (11.8%) were reported.

A total of 1193 individuals were admitted to the three hospitals during the study period. After excluding 11 individuals with missing data, the remaining 1182 participants were included in the analysis (Figure 1). The median age was 86 years (interquartile range: 77-91), and 45.5% were male. A total of 140 falls occurred (11.8%), with an incidence rate of 4.4 per 1000 patient-days. Within the fall group, the median age (interquartile range) was 88 years (83-92), 111 (79.3%) were male, and the median length of hospital stay (interquartile range) was 68 days (32-119) (Table 1).

### Univariate analysis

The results of the univariate analysis are shown in Table 1. Several factors were significantly associated with falls on univariate analysis, including older age (88 years, 95% CI: 83-92 vs. 86 years, 95% CI: 76-79; p <0.001), male sex (79.3% vs. 41.0%), longer hospital stay (68 days, 95% CI: 32-119 vs. 24 days, 95% CI: 11-56; p <0.001), referral status (67.1% vs. 18.6%), hypnotic medication use (55.0% vs. 19.4%), history of falls (90.0% vs. 55.6%), impaired vision (15.0% vs. 9.6%), in-hospital rehabilitation (97.1% vs. 69.7%), absence of emergency admission (44.3% vs. 75.5%) and absence of emergency transport (3.6% vs. 8.6%). Moreover, patients in the fall group were more likely to have BR of A and B and CFS of I, II, III, and M, with different distributions. However, there was no significant difference in eating independence between the fall and non-fall groups.

### Multivariable analysis and performance of predictive models

**Figure 2:**
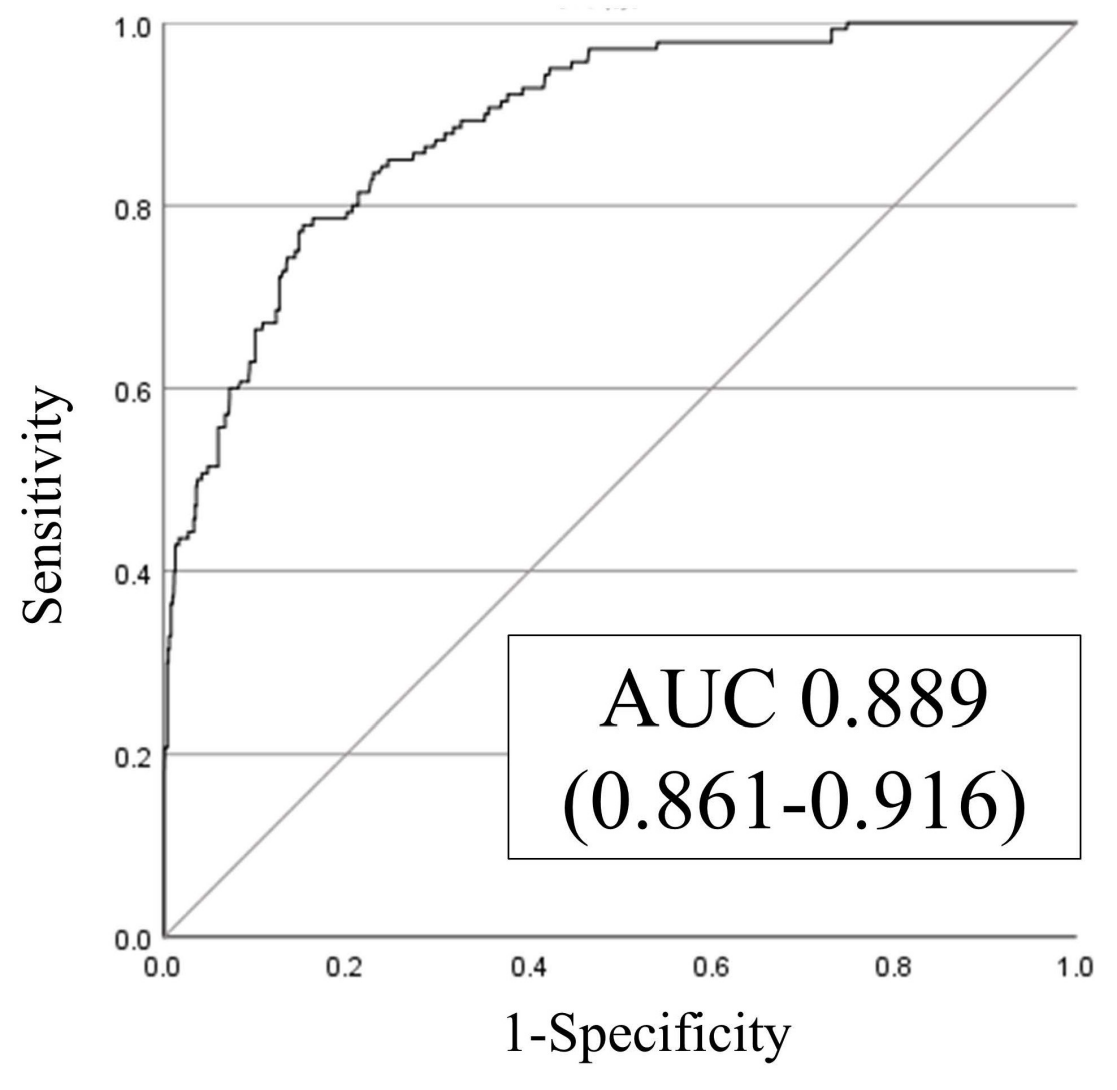
Receiver operating characteristic (ROC) and area under the curve (AUC).

**Figure 3:**
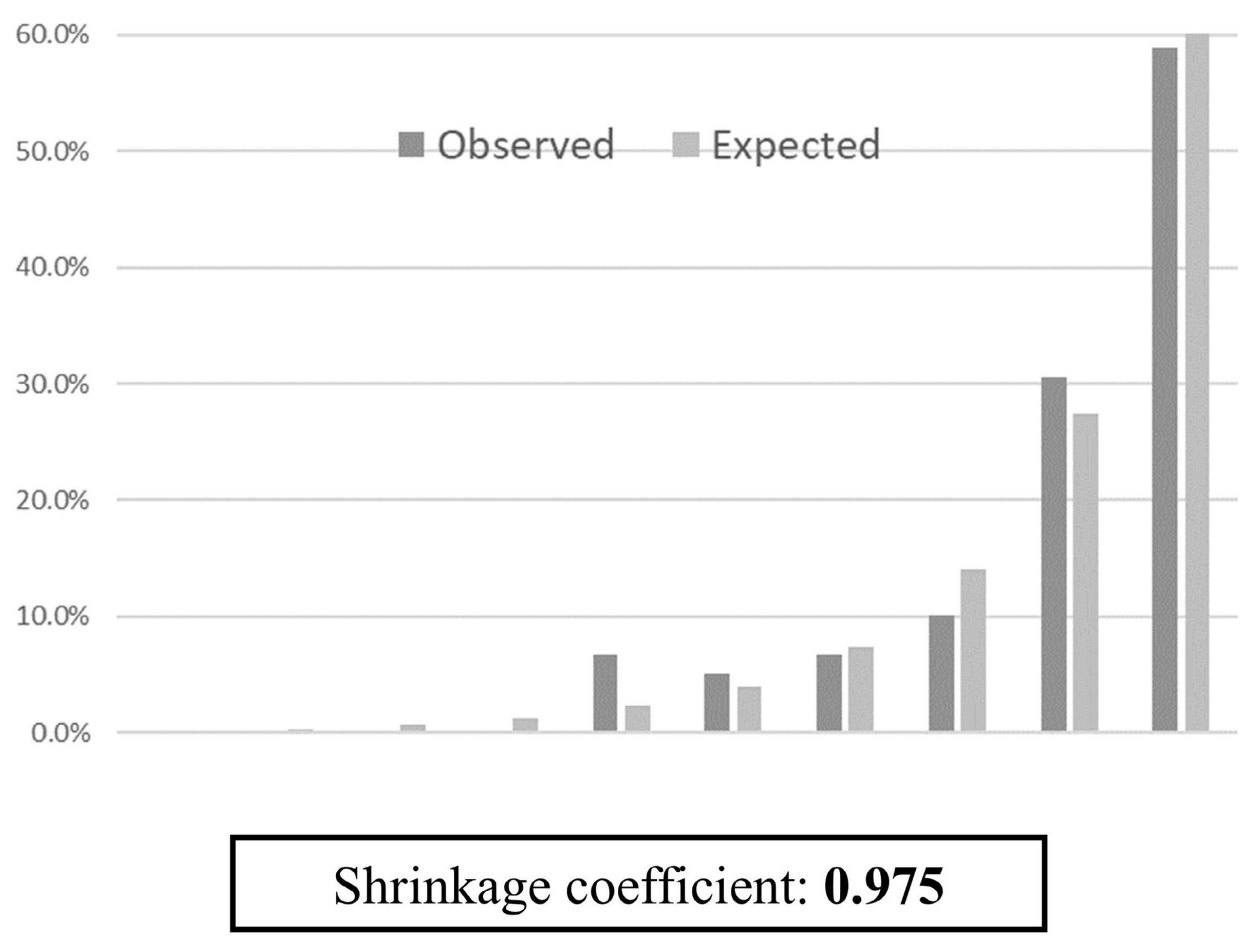
Predicted and observed fall rates in 10 groups divided into deciles by score using SFRM2.

Multivariable logistic regression analysis identified age, male sex, history of falls, emergency admission, hypnotic medication use, eating independence, and BR as significant predictors of in-hospital falls (Table 2). The SFRM2 demonstrated an AUC of 0.889 (95% CI: 0.861-0.916) (Figure 2). The sensitivity, specificity, positive predictive value, and negative predictive value of the model for the different cutoff scores are shown in Table 3. Notably, the observed fall incidence was consistent with the predicted incidence, with a shrinkage coefficient of 0.975 (Figure 3).

## Discussion

This study retrospectively validated the accuracy of SFRM2, our in-hospital fall prediction model developed for acute care, among inpatients at several long-term care hospitals. The AUC of the predictive model was 0.889 (95% CI: 0.861-0.916), indicating high discriminative ability. The shrinkage coefficient of 0.975 and the minimal discrepancy between predictive and observed values further support the high predictive accuracy of SFRM2.

The high AUC for SFRM2 may be attributed to the relatively stable functional status (ADLs and CFS) of inpatients in long-term care hospitals. In this study, most participants presented with severe BR (B: 24.2%, C: 29.9%) and CFS (III: 22.6%, IV: 29.1%), indicating the need for more extensive care and longer length of hospital stay. Studies suggest that ADLs at six months and cognitive impairments remained unchanged in 86.8% of older adults residing in long-term care facilities who require nursing assistance 25. In contrast, approximately 30% of older patients in acute care hospitals experience deterioration in ADLs following discharge 26. Thus, the previous study involving acute care hospital beds with decreased SFRM2 AUCs in the long-term care hospital likely included patients whose ADLs changed during hospitalization 11. Consequently, SFRM2, which relies on admission data, may be more effective in long-term care settings where patient conditions are less likely to change significantly.

The SFRM2, originally developed for acute care hospitals, has demonstrated high predictive accuracy even in long-term care hospitals. Furthermore, its ease of use and effectiveness make it a valuable model in busy clinical settings, contrasting existing fall prediction models that are often complex and time-consuming. Japanese long-term care hospitals are known to provide comprehensive medical, rehabilitation, and long-term care services. While overseas facilities may provide chronic and long-term care, there are no long-term care hospitals equivalent to those in Japan. Additionally, several validations of fall risk models in chronic care hospitals reported limited accuracy. For instance, the HFRM II, widely used in acute care settings, achieved an AUC of 0.72, sensitivity of 85%, and specificity of 43% among patients aged ≥65 years in the geriatric acute care unit of an Italian teaching hospital 14. In a German study of chronic care hospitals for older adults, HFRM II exhibited an AUC of 0.64, with a sensitivity of 75% and specificity of 47% 15. Similarly, the Clinical Frailty Scale demonstrated an AUC of 0.680, with a sensitivity of 44.5% and specificity of 83.6%, in elderly patients in chronic care hospitals 7. However, it should be noted that this scale is subjective, wherein frailty scoring is made based on appearance, interview, and other factors. As such, the evaluation of falls using this subjective assessment tool is difficult. In comparison, SFRM2 offers a more effective and easily applicable approach for predicting falls in both acute care and long-term care hospitals.

Despite the insights offered in this study, several limitations should be acknowledged. First, the retrospective observational design of the study may have affected data accuracy and uniformity. Second, the absence of fall prevention interventions could have influenced our results. Third, this study could not examine potential confounding factors such as medications, environmental factors, and patient background factors. Fourth, sampling variability was observed in the univariate analysis, potentially causing fluctuation in p-values. Future prospective studies considering fall prevention studies are warranted to further validate SFRM2 in long-term care hospitals.

## Conclusion

SFRM2, a simple fall prediction model developed for acute care hospitals, showed good discriminative ability in external validation for predicting falls in long-term care hospitals. Given the relatively stable conditions of older inpatients after admission in Japanese long-term care hospitals, SFRM2 may particularly be beneficial for fall risk assessment in these facilities.

## Supplementary Material

Supplementary information.

## Figures and Tables

**Table 1:** Characteristics of patients and the results of univariate analysis

Variable, Category	All patients n = 1182	Fall Group	Non-Fall Group	p value^†^
		n = 140	n = 1042	
Age, years	86 (77-91)	88 (83-92)	86 (76-91)	< 0.001
Sex, Male	538 (45.5)	111 (79.3)	427 (41.0)	< 0.001
Emergency admission, Yes	849 (71.8)	62 (44.3)	787 (75.5)	< 0.001
Transported by ambulance, Yes	95 (8.0)	5 (3.6)	90 (8.6)	0.038
Referral letter, Presence	288 (24.4)	94 (67.1)	194 (18.6)	< 0.001
Department, Internal Medicine	1165 (98.6)	140 (100)	1025 (98.4)	0.128
Department, others	17 (1.4)	0 (0.0)	17 (1.6)	
Department, Neurosurgery	0 (0.0)	0 (0.0)	0 (0.0)	
Hypnotic medications, Using	279 (23.6)	77 (55.0)	202 (19.4)	< 0.001
History of falls, Presence	705 (59.6)	126 (90.0)	579 (55.6)	< 0.001
Visual impairment, Presence	121 (10.2)	21 (15.0)	100 (9.6)	0.129
Visual impairment, missing	6 (0.5)	1 (0.7)	5 (0.5)	
Eating, Independent	583 (49.3)	78 (55.7)	505 (48.5)	0.107
Eating, Requiring assistance	599 (50.7)	62 (44.3)	537 (51.5)	
Bedriddenness rank, Normal	277 (23.4)	3 (2.1)	274 (26.3)	< 0.001
Bedriddenness rank, J	118 (10.0)	8 (5.7)	110 (10.6)	
Bedriddenness rank, A	147 (12.4)	38 (27.1)	109 (10.5)	
Bedriddenness rank, B	286 (24.2)	69 (49.3)	217 (20.8)	
Bedriddenness rank, C	354 (29.9)	22 (15.7)	332 (31.9)	
Cognitive function score, Normal	333 (28.2)	8 (5.7)	325 (31.2)	< 0.001
Cognitive function score, I	90 (7.6)	12 (8.6)	78 (7.5)	
Cognitive function score, II	123 (10.4)	28 (20.0)	95 (9.1)	
Cognitive function score, III	267 (22.6)	65 (46.4)	202 (19.4)	
Cognitive function score, IV	344 (29.1)	21 (15.0)	323 (31.0)	
Cognitive function score, M	18 (1.5)	6 (4.3)	12 (1.2)	
Cognitive function score, missing	7 (0.6)	0 (0)	7 (0.7)	
Surgical operation, Undergone	1 (0.1)	0 (0)	1 (0.1)	0.714
Rehabilitation, Undergone	862 (72.9)	136 (97.1)	726 (69.7)	< 0.001
Length of hospital stay (days)	27 (12-63)	68 (32-119)	24 (11-56)	< 0.001

^†^p values were calculated by the Mann-Whitney U test for continuous variables and the chi-squared test for categorical variables. Continuous and categorical variables are shown as median value (interquartile range) and number (percent). Bedriddenness ranks: J, independence/autonomy; A, house-bound; B, chair-bound; C, bed-bound. Cognitive function scores: I, almost independent in daily living with only slight cognitive impairment; II, independent with slight difficulty in daily living or communication under careful overseeing; III, dependent in daily living or communication; IV, dependent in daily living or communication, and requires constant care; M, severe psychological symptoms, troubled behaviors or severe physical disorders requiring specialized medical service.

**Table 2:** Result of multivariate logistic regression analysis

	OR	95% Cl	p value^†^
Age	1.1	1.0-1.1	<0.001
Sex, Male (Female)	8.7	5.3-14.4	<0.001
Emergency admission, Presence (Absence)	0.34	0.22-0.55	<0.001
Department, Internal Medicine (Others)	NA	NA	0.998
Hypnotic medications, Using (Not using)	3.6	2.3-5.5	<0.01
History of falls, Presence (Absence)	3.8	1.9-7.6	<0.01
Eating, Independent (Requiring assistance)	2.7	1.6-4.6	<0.001
Bedriddenness rank, J (Normal)	4.2	1.0-17.8	0.050
Bedriddenness rank, A (Normal)	10.1	2.7-38.1	0.001
Bedriddenness rank, B (Normal)	8.8	2.3-33.2	0.001
Bedriddenness rank, C (Normal)	2.7	0.7-11.0	0.161

^†^ p values for Wald test. OR: odds ratio; 95% CI: 95% confidence interval; NA: not available; J: independence/autonomy; A: house-bound; B: chair-bound; C: bed-bound.

**Table 3:** Validation of the predictive model with the cutoff points determined in the present study

Cutoff value for scores	-2.44	-2.14	-2.01
Probability^†^	8.0	10.6	11.8
Sensitivity	90.0	77.9	65.7
Specificity	64.5	84.7	90.0
Positive predictive value	25.4	40.6	46.9
Negative predictive value	98.0	96.6	95.1

^†^ The value was calculated as the probability of a fall for patients with defined scores. Probability=100×Exp(score)/(1+Exp(score))

## Abbreviations

ADLs: activities of daily living
AUC: area under the curve
BR: Bedriddenness rank
CFS: Cognitive function scores
CI: confidence interval
HFRM II: Hendrich II fall risk model
MHLW: Ministry of Health, Labour and Welfare
SFRM2: Saga fall risk model 2
