# Supplementary material for: Validation of a Fall Predictive Model for Inpatients in Japanese Long Term Care Hospitals
Source: Int J Med Sci. 2025 Jun 9;22(12):2877–83. doi: 10.7150/ijms.106600 (PMC12243853; doi:10.7150/ijms.106600)
Supplement: Supplementary file 1 — Supplementary information. [file ijmsv22p2877s1.pdf]

Supplementary material
S1. Characteristics of the three hospitals

Isumi Medical Center

The hospital has 12 departments with 144 beds, of which 70 are for acute care, 48 for long-term care, 22 for community-based care, and 4 for infectious disease. The departments include Internal Medicine, General Surgery, Gynecology, Ophthalmology, Otorhinolaryngology, Uranology, Neurology, Breast surgery, Pediatrics, Dermatology, and Orthopedic Surgery. The hospital is located in Isumi City, Chiba, Japan, covering an approximate population of 35,600, and functions as a center for the integrated community care system from acute to long-term care.

Karatsu Municipal Hospital

The hospital provides outpatient care in Internal Medicine, General Surgery, Rehabilitation, Orthopedics Surgery, and Pediatrics. The hospital contains 56 long-term care beds and focuses on rehabilitation. It is located in Karatsu City, Saga, Japan, covering an approximate population of 117000.

Shimada Hospital of Medical Corporation Chouseikai

The hospital provides outpatient care in Internal Medicine and Dental services, and has 84 long-term care beds for patients requiring high nursing care. The hospital is located in Saga City, Saga, Japan, with a population of 232,000.
